# Experience Sampling as a dietary assessment method: a scoping review towards implementation

**DOI:** 10.1186/s12966-024-01643-1

**Published:** 2024-08-27

**Authors:** Joke Verbeke, Christophe Matthys

**Affiliations:** 1 Clinical and Experimental Endocrinology, Department of Chronic Diseases and Metabolism, KU Leuven, Leuven, Belgium; 2 Department of Endocrinology, University Hospitals Leuven, Leuven, Belgium

**Keywords:** Ecological Momentary Assessment, Nutrition Assessment, Mobile Health, Food Frequency Questionnaire, Epidemiology, Food Diary

## Abstract

**Background:**

Accurate and feasible assessment of dietary intake remains challenging for research and healthcare. Experience Sampling Methodology (ESM) is a real-time real-life data capturing method with low burden and good feasibility not yet fully explored as alternative dietary assessment method.

**Methods:**

This scoping review is the first to explore the implementation of ESM as an alternative to traditional dietary assessment methods by mapping the methodological considerations to apply ESM and formulating recommendations to develop an Experience Sampling-based Dietary Assessment Method (ESDAM). The scoping review methodology framework was followed by searching PubMed (including OVID) and Web of Science from 2012 until 2024.

**Results:**

Screening of 646 articles resulted in 39 included articles describing 24 studies. ESM was mostly applied for qualitative dietary assessment (i.e. type of consumed foods) (*n* = 12), next to semi-quantitative dietary assessment (i.e. frequency of consumption, no portion size) (*n* = 7), and quantitative dietary assessment (i.e. type and portion size of consumed foods) (*n* = 5). Most studies used ESM to assess the intake of selected foods. Two studies applied ESM as an alternative to traditional dietary assessment methods assessing total dietary intake quantitatively (i.e. all food groups). ESM duration ranged from 4 to 30 days and most studies applied ESM for 7 days (*n* = 15). Sampling schedules were mostly semi-random (*n* = 12) or fixed (*n* = 9) with prompts starting at 8–10 AM and ending at 8–12 PM. ESM questionnaires were adapted from existing questionnaires, based on food consumption data or focus group discussions, and respond options were mostly presented as multiple-choice. Recall period to report dietary intake in ESM prompts varied from 15 min to 3.5 h.

**Conclusions:**

Most studies used ESM for 7 days with fixed or semi-random sampling during waking hours and 2-h recall periods. An ESDAM can be developed starting from a food record approach (actual intake) or a validated food frequency questionnaire (long-term or habitual intake). Actual dietary intake can be measured by ESM through short intensive fixed sampling schedules while habitual dietary intake measurement by ESM allows for longer less frequent semi-random sampling schedules. ESM sampling protocols should be developed carefully to optimize feasibility and accuracy of dietary data.

**Supplementary Information:**

The online version contains supplementary material available at 10.1186/s12966-024-01643-1.

## Background

Research on health and nutrition relies on accurate assessment of dietary intake [1]. However, dietary intake is a complex exposure variable with high inter- and intra-variability existing of different components ranging from micronutrients, macronutrients, food groups, meals to the dietary pattern as a whole. Therefore, measuring dietary intake accurately and feasibly is challenging for both researchers and healthcare professionals [2–4]. Only few established nutritional biomarkers are available and, therefore, no objective method exist to reflect true dietary intake or the dietary pattern as a whole in epidemiological research [2, 3]. Instead, most dietary assessment methods rely on self-report. Food records, referred to as the “golden standard”, together with 24-h dietary recalls provide most detailed dietary data while Food Frequency Questionnaires (FFQ) reflects habitual (i.e. long-term usual intake) dietary intake which is the variable of interest in most diet-disease research [4–6]. Food records, 24-h dietary recalls, and FFQs have known limitations and challenges including recall bias, social-desirability bias, misreporting, and burdensomeness contributing to inherent measurement error in dietary intake data [2, 6]. A review of Kirkpatrick et al*.* showed that feasibility, including cost-effectiveness and ease-of-use, is the main determinant for researchers in selecting a dietary assessment method instead of appropriateness for study design and purpose at the expense of data quality and accuracy [7]. To advance nutritional research and enhance the quality of dietary data, exploring the implementation of new methodologies is warranted to improve feasibility and overcome the limitations of current dietary assessment methods.

Experience Sampling Methodology (ESM), an umbrella term including Ecological Momentary Assessment (EMA), ambulatory assessment, and structured diary method, refers to intensive longitudinal assessment and real-time data-capturing methods [8]. Participants are asked to respond to short questions sent through smartphone prompt messages or beeps at random moments during the day to assess experiences or behaviors and moment-to-moment changes in daily life [9]. Originating from the field of psychology and behavioral sciences, ESM typically assesses current mood, cognitions, perceptions, or behaviors and descriptors of the momentary context (i.e. location, company) [9]. Usually, assessments are collected in a random time sampling protocol yet, assessments can also be triggered by an event (event-contingent sampling), at fixed time points, or random within fixed time intervals (semi-random). ESM questionnaires are usually designed to be completed in under 2 min consisting of open-ended questions, visual analogue scales, checklists, or self-report Likert scales. Several ESM survey applications (i.e. m-Path, PsyMate, PocketQ) are currently available in which the sampling protocol and questionnaires can be customized to the study design and aim [10, 11]. It was shown that ESM reduces recall bias, reactivity bias, and misreporting in psychology and behavioral research by its design through unannounced, rapid, real-life, real-time repeated assessments [12]. For this reason, Experience Sampling might be an interesting new methodology to explore as an alternative dietary assessment methodology. The design of ESM could overcome recall bias, reactivity bias, social desirability bias, and misreporting seen in traditional dietary assessment methods. However, the application of ESM for dietary assessment is new. Defining and balancing ESM methodological considerations, i.e. study duration, frequency and timing of sampling (signaling technique), formulation of questions and answer options, is a delicate matter and crucial in balancing feasibility with data accuracy [13].

The application of ESM in the field of dietary assessment has not been fully explored yet. Schembre et al*.* reviewed ESM for dietary behavior for the first time [12]. However, it has not yet been assessed how ESM could be implemented as an alternative dietary assessment method aiming to estimate daily energy, nutrient, and food group intake quantitatively.

Therefore, this scoping review investigates how Experience Sampling Methodology can be implemented to develop an Experience Sampling-based dietary assessment method as an alternative to traditional dietary assessment methods to measure daily energy, nutrient, and food group intake quantitatively. This review aims to map ESM sampling protocols and questionnaire designs used to assess dietary intake. Additionally, the findings of this review will be combined with best practices to develop ESMs and dietary assessment methods to formulate key recommendations for the development of an Experience Sampling-based Dietary Assessment Method (ESDAM). The following questions will be answered:

i) How is ESM applied in literature to assess dietary intake - focusing on methodological considerations (i.e. development and formulation of questions and answers, selection and consideration of prompting schedule (timing and frequency))?
ii) How can ESM specifically be applied for quantitative assessment of total dietary intake (i.e. as an alternative to traditional dietary assessment method)?

## Methods

### Design

This scoping review followed the methodological framework for scoping reviews of Arksey and O’Malley which was further developed by Levac et al. [14, 15]. A scoping review approach was chosen to explore and map the design aspects and considerations for developing experience sampling methods to assess dietary intake as an alternative to traditional dietary assessment methods, which is novel. Moreover, this review will formulate design recommendations to apply ESM as a dietary assessment method and will serve as starting point to develop an ESDAM. An a priori protocol was developed based on the Preferred Reporting Items for Systematic review and Meta-Analysis Protocols (PRISMA-P) and the Joanna Briggs Institute Scoping Review protocol template (Supplementary Material) [16, 17]. According to Arksey and O’Malley methodological framework, the iterative nature of scoping reviews may include further refinement of the search strategy and the inclusion and exclusion criteria during the initial review process due to the unknown breadth of the topic [14]. Therefore, adaptations made to the methodology described in the a priori protocol based on initial searches are described below. This scoping review was reported according to the PRISMA extension for scoping reviews (PRISMA-ScR) [18].

### Search strategy and screening

The search strategy was developed based on key words and Mesh terms for “dietary assessment” and “experience sampling” (Supplementary Material). The term “ecological momentary assessment” was included as a synonym of ESM. Electronic databases PubMed (including MEDLINE) and Web of Science were searched for relevant literature published between January 2012 and February 9th 2024. The year 2012 was chosen as lower limit for inclusion since this review focuses on the use of ESM by digital tools (i.e. smartphones, web-based or mobile applications) which has emerged especially since the introduction of applications for smartphones since 2008. Therefore, the time frame of this review is focused on literature published in the last 12 years. The reference lists of all included articles were screened for additional studies.

The initial search strategy described in the protocol was developed based on the assumption that research using ESM as an alternative to traditional dietary assessment was limited. Therefore, initially, research using ESM in the broader field of health research was included to obtain more evidence on methodological considerations of application of ESM. In line with the Arksey and O’Malley methodological framework, inclusion criteria were adapted following initial searches along with discussion and consensus between the reviewer (JV) and principal investigator (CM). Therefore, inclusion criteria were adapted to research applying ESM to measure dietary intake quantitatively or qualitatively since literature was also available in the field of dietary behaviour in relation to contextual factors (Table 1). Studies measuring dietary behaviour (i.e. cravings, hunger, eating disorder behaviour, dietary lapses) only, without assessing dietary intake, were excluded. Event-based ESM as dietary assessment method was excluded since this was deemed a similar methodology as the food record and, therefore, not serving the purpose of this review to explore a new methodology for dietary assessment to overcome limitations of traditional dietary assessment methods. All inclusion and exclusion criteria are presented in Table 1.

**Table 1:** Inclusion and exclusion criteria of the scoping review adapted from the protocol following initial searches

Inclusion	Exclusion
1) Studies using ESM tools for dietary assessment 2) Studies on methodological aspects of developing ESM tools measuring dietary intake	1) Studies not assessing dietary intake 2) Studies using ESM to measure dietary intake based an event-contingent approach only 3) Studies using ESM not through a mobile device (i.e. paper and pencil) 4) Reviews, case reports, conference abstracts, editorial and opinion pieces, book reviews, and book synopses 5) Non-English studies 6) Articles of which full text is not available 7) Animal studies

All records were exported and uploaded into the review software Rayyan. Duplicates were identified through the software followed by a manual screening of the reviewer for confirmation and removal of duplicates. One reviewer (JV) screened the retrieved articles first by title and abstract followed by a full text screening [19–21]. In case of hesitancy on inclusion of articles, the reviewer (JV) consulted the principal investigator (CM) to reach consensus. In line with established scoping review methods, methodological quality assessment was not performed [14, 18]. Since this review aims to shed light on design aspects and considerations of ESM and, thus focuses on the application of the methodology used in the articles rather than the study outcome, quality assessment was considered not relevant for this purpose.

### Data extraction

Data were extracted in an Excel table describing the authors, title, year of publication, signalling technique, timing of prompts, study duration, dietary variables measured, answer window, (formulation of) questions, respond options, notification method, indication of qualitative or quantitative dietary assessment, delivery method, population and study name. All data were described qualitatively. Studies applying ESM for dietary assessment were categorized in separate tables for ESM used for qualitative dietary assessment (i.e. assessment of type of foods consumed without portion size, not allowing estimation of nutrient intake), ESM used for semi-quantitative dietary assessment (i.e. assessment of type of foods or frequency of consumption of foods, not allowing estimation of nutrient intake), and ESM used for quantitative dietary assessment (i.e. assessment of type of foods consumed and portion size, allowing estimation of nutrient intake).

## Results

### Literature search and study characteristics

**Fig. 1:**
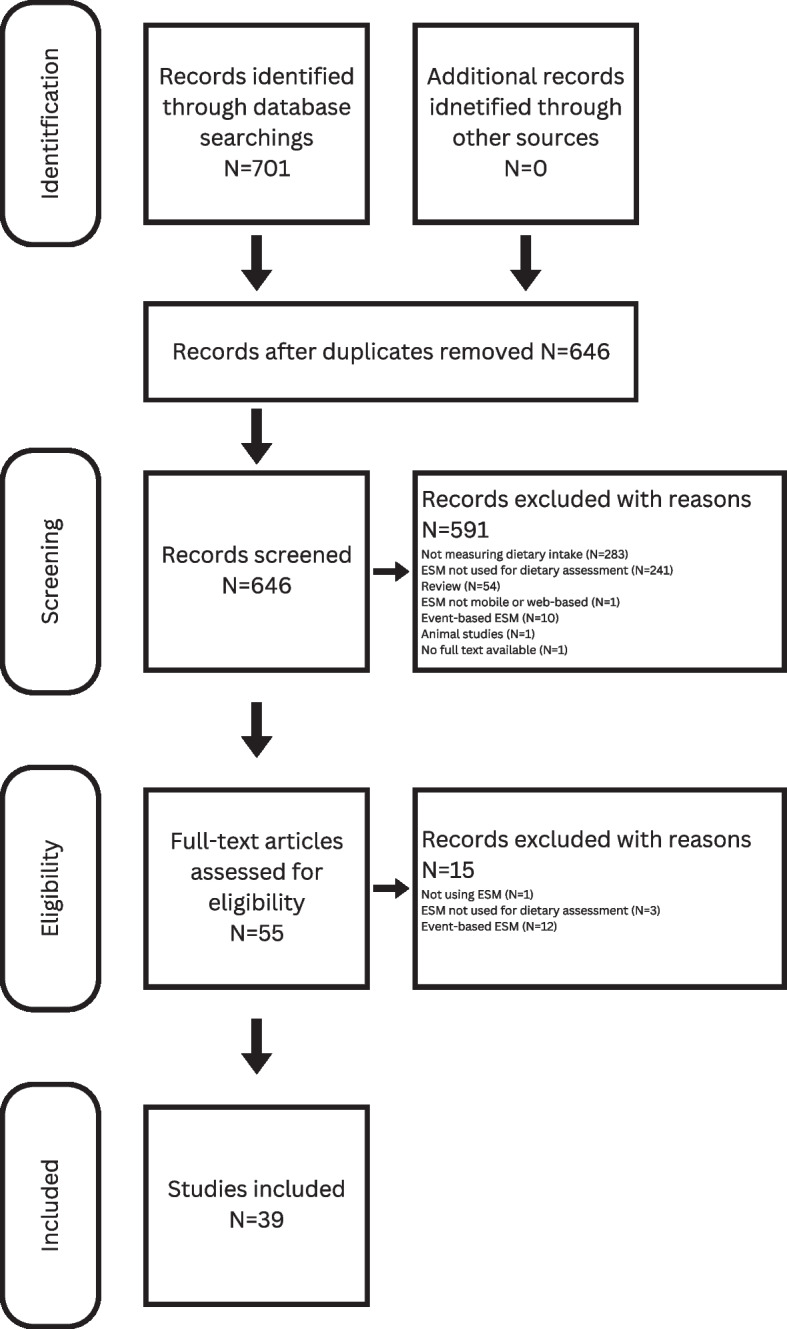
PRISMA flow diagram of the screening and selection process

The electronic databases search resulted in 701 articles of which 55 duplicates were identified and removed. Next, 646 articles were screened by title and abstract of which 591 were excluded according to the exclusion criteria (Fig. 1). The remaining 55 articles were screened by full text. After exclusion of 16 articles following full text screening, 39 articles were selected for inclusion (Table 2). The included articles describe 24 individual studies of which the Mother’s and Their Children’s Health (MATCH) study was described most frequently (*n* = 12, 25%). Most studies were published in 2018 (*n* = 7), followed by 2020 (*n* = 6) and 2022 (*n* = 6). Students, including both high school and higher education students, were the study population in most EMA or ESM studies included (*n* = 10, 43%). Two studies applied the ESM methodology to assess dietary behaviour including dietary variables of children with mothers as proxy. Five studies referred to their methodology using the terminology ‘ESM’ while the other studies used ‘EMA’ as terminology.

**Table 2:** Methodological considerations of included studies^a^ which applied ESM for qualitative dietary assessment

Author	ESM Sampling scheme			ESM questionnaire design				
	**Signaling technique**^**b**^	**ESM duration**	**Frequency and timing of prompts**	**Dietary variable** **assessed**	**Questions**	**Respond options**	**Rationale for questionnaire design**	**Respond window**
[22]	SR, EC, EOTDQ	7 days	EC: self-initiated at each eating event; SR: 2 random prompts on school days (15 h-18 h, 18 h-21 h), 4 random prompt on non-school days (9 h-12 h, 12 h-15 h, 15 h-18 h, 18 h-21 h); EOTDQ: 6 h-23h45	Food and beverage intake, focus on snacks and sweetened beverages	“Were you eating or drinking anything?”	Yes/no respond option. If answer is yes, multiple choice option to select what they were eating: (1) drink only, (2) snack with or without a drink, and (3) meal with or without a drink	Partially based on information collected from focus groups with adolescents to learn about their eating patterns, with a special focus on snacking (locations, social environment, types of snacks and drinks, etc.), mood scales, and food-related cues such as the sight or smell of food identified in studies on restrained eating	Not specified
[23, 24], [25, 26], [27], [28–30], [31, 32], [33]	SR	8 days	SR: 3 times on weekdays, 7 times weekend days (1 per time window) weekdays: 15h30-16 h, 17h30-18 h, 19h30-20 h; weekend days: 7h30-8 h, 9h30-10 h, 11h30-12 h, 13h30-14 h, 15h30-16 h, 17h30-18 h, 19h30-20 h	Fruit and vegetables, fries or chips, sweets and pastries, soda and energy drink consumption	First prompt of the day: ‘Since you woke up this morning, which of the following have you done? (Choose all that apply)’ Every following prompt: ‘In the past 2 HOURS, which of the following have you done? (Choose all that apply)’	Multiple choice: ‘Eaten Fruits or Vegetables’, ‘Eaten Chips or Fries’, ‘Eaten Pastries or Sweets’, ‘Drank Soda or Energy Drinks (not counting diet)’ and ‘None’	Not specified	Not specified
[34]	R	4 days	2 times per day during each of the four established time periods: 9h00-12h00, 12h00-15h00, 15h00-19h00, 19h00-22h00	Consumption of (1) sweets; (2) salty foods; (3) fruits and vegetables; (4) entrées, (5) breads and grains	Real-time prompts asked participants what they were doing in the moment before they received the prompt and retrospective prompts asked participants to recall what they did in the past 3 h	Multiple choice: ‘eating’, ‘drinking’, ‘being physically active’, or ‘none of the above’ If ‘eating’ was selected, following answer options were: (1) sweets, (2) salty snacks/fried side dishes, (3) fruits and vegetables, (4) entrées (eg, pizza, sandwiches, lasagnas, chicken), (5) breads/grains, and (6) other	Not specified	35 min (5 min prior to prompt, 30 min after)
[35]	SR	30 days	3 times a day in time windows: 7h00-10h00, 14h00-17h00, 17h00-24h00	Unhealthy food consumption	Daily consumption of following foods was examined: ‘fast food’, ‘caffeinated drinks’ (including soda and sweet tea), ‘not consuming any fruit’, and ‘not consuming any vegetables’	Yes/no respond option to indicate (missing) consumption of the proposed foods that day	Not specified	Not specified
[36]	R	8 days	Up to 8 times per day between 8h30-20h30	Snacks and SBB consumption	"Is your child eating any of the following?" (fruit and vegetable consumption), "What is your child eating?" (snack consumption), "What is in the cup or bottle?" (SSB consumption)	No fruit or vegetables: (i) cookies or other sweet foods/desserts; (ii) chips or other salty foods; (iii) French fries, chicken nuggets or other fried foods; (iv) none of the above. Fruits or vegetables: (i)fruit; (ii)vegetables (not including French fries); Snacks: meal or snack; SSB: no SSB: (i) whole milk; (ii)low-fat or skimmed milk, (iii) breast milk/formula, (iv) water, (v) diet soda or other diet drink, (vi) juice, 100% or SSB: (i) juice or other sweetened drink (like iced tea or Kool Aid)	Not specified	15 min
[37]	SR	4 days	Up to 15 times per day (1 every hour) during (self-reported) waking time	Highly processed food intake and palatable food intake	“In the last hour, did you …” for the following: “eat sweet high-fat foods (e.g., cookies) “eat fast foods?” and, “drink non-alcoholic sugary drinks?” “In the last hour, did you eat palatable foods (e.g., food that is most pleasurable to you)?”	Yes/no respond option	The specific food/drink groups were selected based on prior literature indicating reward processes are implicated in intake of these foods and because greater proportional intake of these foods can damage physical health ‘Palatable foods’ was selected based on long-standing literature indicating that unique foods are pleasurable to each individual	Not specified
[38]	SR	7 days	5 times per day, 1 h segments (i.e., 9h30–10h30, 12h30–13h30, 15h30–16h30, 18h30–19h30, 21h30–22h30)	Fruits, vegetables, fast food, chips or fries, pastries or sweets, and whole wheat foods, water and SBB	“Did you eat within the past 2 h?” If so, a follow-up question asked participants to indicate the types of foods consumed within the past 2 h. “Had you anything to drink within the past 2 h?”	Yes/no respond option. If answer is yes, type of food needed to be indicated: ‘fruits,’ ‘vegetables,’ ‘fast food,’ ‘chips or fries,’ ‘pastries or sweets,’, and ‘whole wheat foods’, ‘water,’ ‘soda or sweet tea,’ ‘diet soda,’ ‘energy drink,’ ‘sports drink,’ ‘coffee,’ ‘milk,’ ‘fruit juice,’ and ‘alcohol.’	Not specified	15 min, reminder every 5 min
[39]	SR	14 days	3h00-9h00; 9h00-15h00; 15h00-21h00; 21h00-3h00 (study population: night nurses)	Empty calorie food/beverage consumption	“Since the last signal, have you consumed or used any of the following items? (Please check all that apply).”	Multiple choice respond option of 21 food/beverage items which were grouped into the following categories: fried/fast food, sweet snack foods (ie, candy), salty snacks (eg, chips), and sugar-sweetened beverages	Food items in the checklist based on the top sources of empty calorie foods/beverages reported in the 2003–2006 National Health and Nutrition Examination Survey (NHANES), the 1993–1996 Nutrition and Health Survey in Taiwan (NAHSIT), and the 2005–2008 NAHSIT. Based on the food/beverage items listed in the food frequency questionnaire employed in the NAHSIT	1 h, reminder every 15 min
[40]	F	7 days	3 times per day following customized schedules coinciding with their regular meal-times (median time for breakfast: 0915 h; lunch: 1245 h; dinner: 1845 h)	Meal consumption	At each prompt, participants were asked: (i) whether they had eaten in the past 15 min (yes/no), (ii) whether they had used their phone during the meal (yes/no), and (iii) what phone functions they had used (free response question). As part of a larger study, they also addressed questions about their food consumption patterns	Yes/no respond option	Not specified	30 min
[41]	SR	7 days	Within 2-h time window around five anchor times: 9:00am, 11:00am, 2:00 pm, 5:00 pm, and 8:00 pm; and morning survey	Meal consumption	Report if a meal or snack was eaten since the last survey and note the approximate times of each meal	Yes/no respond option	Not specified	60 min with reminder after 30 min
[42]	F, EC	7 days	4 times per day: 9h00, 13h00, 17h00, 21h00	Consumption of high-fat foods	“Have you eaten any food since the previous prompt?”	Yes/no respond option. If the subject replied “yes”, a list of high-fat foods was presented to select all the food items that applied	Not specified	Not specified
[43]	SR	7 days	5 times per day (07h00–10h00, 10h00–13h00, 13h00–16h00, 16h00–19h00, 19h00–22h00)	Foods and beverages consumed during the day (i.e., at breakfast, morning and afternoon snack, lunch, and dinner) from a list	“Select if the following 35 food items were eaten or not”	Multiple choice respond option: ‘breakfast cereals’, ‘biscuits’, ‘white bread’, ‘wholemeal bread’, ‘pasta’, ‘rice’, ‘vegetables’, ‘legumes’, ‘soup’, ‘fruit’, ‘nuts’, ‘red meat’, ‘white meat’, ‘processed meat’, ‘offal, fish’, ‘canned fish’, ‘shellfish’, ‘curd cheese’, ‘ripened cheese’, ‘eggs, milk’, ‘yoghurt’, ‘pizza’, ‘salty snacks’, ‘fries’, ‘sweets and refined sugar’, ‘olive oil’, ‘vegetable oil’, ‘butter and margarine’, ‘dipping sauces’, ‘energy drinks’, ‘fruit juice’, ‘coffee’, ‘tea’, ‘wine’, ‘beer’, ‘spirits’	Not specified	Not specified

^a^ Manuscripts describing research based on the same study protocol were aggregated and described as one ESM study protocol in the table

^b^ Signalling techniques commonly applied in Experience Sampling protocols:

*R* Random signalling: completely random timing of prompt messages between pre-defined (waking) hours, *F* Fixed signalling: prompt messages sent at predefined time(s), *SR* Semi-random signalling: prompt messages sent at random moments during predefined time windows, *EC* Event contingent sampling: self-initiated registering/responding when predefined event occurs, *EOTDQ* End-of-the-day-questionnaire: questionnaire sent at the end of the day at predefined time (often in combination with signalling  techniques)

### Application of ESM for dietary assessment in literature

#### Dietary variables measured through ESM

Most studies assessed consumption of specific foods only [22–39, 42, 44–55]. Table 2, 3 and 4 provide an overview of the included studies described in the manuscripts with description of specific ESM methodology characteristics according to qualitative, semi-quantitative and quantitative dietary assessment respectively. Four studies used ESM to assess snack consumption [45–51]. Four studies focused on snack and sugar sweetened beverage (SBB) consumption only [22, 36, 44, 52, 53]. Piontak et al*.* applied ESM to assess unhealthy food consumption including fast food, caffeinated drinks and not consuming any fruit or vegetables [35]. Two studies focused on palatable food consumption of which the study of Cummings et al*.* assessed palatable food consumption together with highly processed food intake [37, 54]. Lin et al*.* applied ESM to measure empty calorie food and beverage consumption while Boronat et al*.* assessed Mediterranean diet food consumption [39, 55]. Two studies assessed the occurrence of food consumption only without assessing type of foods consumed [40, 41]. The study of de Rivaz et al*.* assessed the largest type of meal consumed in between signals [56]. Three studies aimed to assess total dietary intake of which the study of Lucassen et al*.* evaluated approaches to assess both actual and habitual dietary intake using ESM [43, 57–59].

**Table 3:** Methodological considerations of included studies^a^ which applied ESM for semi-quantitative dietary assessment

Author	ESM Sampling scheme			ESM questionnaire design				
	**Signaling technique**^**b**^	**ESM duration**	**Frequency and timing of prompts**	**Dietary variable** **assessed**	**Questions**	**Respond options**	**Rationale for questionnaire design**	**Respond window**
[44]	SR	7 days	Within 5 time windows per day (8h00, 12h00, 15h30, 18h30 and 21h30 with a range of 30 min each), tailored to the schedule of their schools	Snacks and sugar sweetened beverages (SBB) consumption	“In the last three and a half hours, what type of snack(s) did you eat? And how many?”; “In the last three and a half hours, have you eaten fruit or vegetables?”	Multiple-choice bullets as response categories for daily meal consumption. Multiple-choice open field combined with VAS sliders to indicate type and frequency of fruits and vegetables, snacks, and sodas consumed. Sliders ranged from 0–100 with open fields enabling participants to fill in amounts ˃100. Binary response categories were used for binary questions	Questions were adapted from a validated questionnaire	60 min, reminder after 30 min
[52]; [53]	SR	7 days	5 times per day in time windows: 7h00-10h00, 10h00-13h00, 13h00-16h00, 16 h-19h00, 19 h-22 h	Snack and sweetened beverage consumption	“Did you consume any of the following foods or beverages since the last signal: i) French fries or other fried side dish, ii) salty snacks such as potato chips, iii) cookies or sweetened baked good (e.g., cake, donut, cookie), iv) chocolate or candy, v) ice-cream or frozen dessert, and vi) sweetened beverage (e.g., pop, juice)?	Yes/no respond option, each positive response for items i-v was summed and dichotomized as none (0) or one, or more than one (> = 1) snack food item	This checklist was adapted from the Dietary Screener Questionnaire (DSQ) (National Health Interview Survey, 2014)	Not specified
[49]	F	7 days	2 times per day at 14h00 and at 22h00	Salty snack consumption	14 h prompt: question one: salty snack food intake between the previous night's prompt and sleep (i.e., from 10:00 p.m. until falling asleep), question two: salty snack food intake between waking up and the current time (i.e., snacks eaten throughout the day until 2:00 p.m.). 22 h prompt: salty snack food intake between the previous prompt and the current time (i.e., 2:00 pm and 10:00 pm)	Multiple choice options which specified different sizes of snack servings (i.e., 0, 1, 2–3, or 4 or more servings), each of which was presented using common equivalents of the specified serving size, e.g., “one snack size bag of Doritos or Tortilla chips.”	Not specified	Answering possible till next prompt, reminder after 90 min
[50]	F	7 days	5 times a day: 10h00, 13h00, 16h00, 19h00, 22h00	Snack consumption	“How many snacks did you consume since the last signal”	Number of consumed snacks	Not specified	60 min, every 10 min reminder
[55]	R	7 days	4 per day at 21 h	Mediterranean diet food consumption	Intake of specified foods during last 24 or 48 h	1. extra virgin olive oil (yes/no), 2. vegetables (n° servings), 3. fruit (n° servings), 4. whole-grain food, 5. sugary drinks (including juices) (n° servings), 6. legumes (n° servings), 7. nuts (n° servings), 8. sweets (yes/no), 9. fish and sea food (n° servings), 10. red meat (n° servings), 11. processed meat (n° servings)	Not specified	12 min
[54]	F	20 days	Once per day: end of the day	Palatable food consumption	Question on number of palatable foods of each food category (sweet, starchy, fast, fatty) consumed during the day	Open field	Examples of foods in each category were provided from the highest factor loadings on each construct from the Food Craving Inventory questionnaire	Not specified
[56]	F	7 days	4 times per day: 8h00, 12h00, 16h00, 20h00	Dietary intake	Question on foods consumed since last assessment	If participants endorsed food consumption since the last assessment, they were asked to indicate the largest quantity of food consumed during this period (snack, small meal, medium meal, or large meal)	Not specified	30 min

^a^ Manuscripts describing research based on the same study protocol were aggregated and described as one ESM study protocol in the table

^b^ Signalling techniques commonly applied in Experience Sampling protocols:

*R* Random signalling: completely random timing of prompt messages between pre-defined (waking) hours, *F* Fixed signalling: prompt messages sent at predefined time(s), *SR* Semi-random signalling: prompt messages sent at random moments during predefined time windows, *EC* Event contingent sampling: self-initiated registering/responding when predefined event occurs, *EOTDQ* End-of-the-day-questionnaire: questionnaire sent at the end of the day at predefined time (often in combination with signalling  techniques)

**Table 4:** Methodological considerations of included studies^a^ which applied ESM for quantitative dietary assessment

Author	ESM Sampling scheme			ESM questionnaire design				
	**Signaling technique**^**b**^	**ESM duration**	**Frequency and timing of prompts**	**Dietary variable** **assessed**	**Questions**	**Respond options**	**Rationale for questionnaire design**	**Respond window**
[45–48]	SR	7 days	Between 7h30-22h30: 10 times per day, start-of-the day and end-of the day questionnaire	Snack consumption	Start-of-the day and end-of the day questionnaire: 37 item questionnaire (5 min). Random prompts: “Did you eat or drink anything between meals since the last beep?”	Yes/no respond option, if yes every product and quantity needed to be specified by a built-in search function based on the Dutch Food Composition Database. For every reported snack, participants chose between two quantity options: standardised quantities (i.e. one apple, one Mars candy bar) or in grams/milliliters. Products with undetermined quantities such as yoghurt and tea could be reported in relevant household measurements (i.e. a bowl or a cup) or in grams/milliliters. Products which were not available in the search function could be added manually	Not specified	Not specified
[51]	F	7 days	5 times per day: 10h00; 13 h; 16 h; 19 h; 22 h	Chocolate snack consumption	Number of consumed snacks since the last prompt and indication of the type of snack they had consumed in a text box	Possibility to register number of snacks consumed with open text field to indicate type of snack	Not specified	Not specified
[58]	F	6 days	6 times per day, every 2.5 h between 9h30-22h00	Food consumption	Participants indicated whether or not they had eaten in last 2.5 h	If participants reported they had eaten, they were prompted to provide a detailed list of foods eaten and the amount in a text box with no word limit. At the study’s in-person information session, participants were instructed to be as detailed as possible regarding what they ate and the amount	Not specified	30 min
[57, 59]	Actual intake: SR; habitual inake: R	4 weeks	Actual intake: 2 week days & 1 weekend day spread over 4 weeks: every 2-h and one morning question on previous night, habitual intake: 7 times per week during 4 weeks	Quantitative dietary intake, meal time (breakfast/lunch/dinner/snack)	Food intake during last 2 h	Subjects could choose from an extensive food list, based on the Dutch Food Composition Database; Amount was reported in household measures (e.g., cups, spoons), standard portion size (e.g., small, large) or amount in gram	Not specified	60 min. In reality, the 2hRs remained open until the end of the day
[56]	F	7 days	4 times per day: 8h00, 12h00, 16h00, 20h00	Quantitative dietary intake	Food consumed since last assessment	If participants endorsed food consumption since the last assessment, they were asked to indicate the largest quantity of food consumed during this period (snack, small meal, medium meal, or large meal)	Not specified	30 min

^a^ Manuscripts describing research based on the same study protocol were aggregated and described as one ESM study protocol in the table

^b^ Signalling techniques commonly applied in Experience Sampling protocols:

*R* Random signalling: completely random timing of prompt messages between pre-defined (waking) hours, *F* Fixed signalling: prompt messages sent at predefined time(s), *SR* Semi-random signalling: prompt messages sent at random moments during predefined time windows, *EC* Event contingent sampling: self-initiated registering/responding when predefined event occurs, *EOTDQ* End-of-the-day-questionnaire: questionnaire sent at the end of the day at predefined time (often in combination with signalling  techniques)

#### Qualitative versus quantitative dietary assessment through ESM

As shown in Table 2, twelve studies performed qualitative dietary assessment (i.e. assessing type of foods consumed without quantification) (Table 2). Seven studies performed semi-quantitative dietary assessment (i.e. assessing frequency of meals/eating occasions or number of servings of food categories not allowing nutrient calculation) [44, 49, 50, 52–56] (Table 3). Quantitative dietary assessment, in line with the aim of traditional dietary assessment methods (i.e. assessment of both type and quantity of foods consumed allowing to estimate nutrient intake), was performed in four studies of which Wouters et al*.* and Richard et al*.* assessed snack intake only while Jeffers et al*.* and Lucassen et al*.* assessed overall dietary intake (i.e. all food groups) [45–48, 51, 57, 58] (Table 4).

#### Study duration, ESM timing and signaling technique

Study duration of ESM dietary assessment varied from four to thirty days of which most studies (*n* = 15) had a study duration of seven days of ESM dietary assessment. The study of Piontak et al*.* had the longest duration of 30 days of ESM assessment [35]. The semi-random sampling scheme (i.e. random sampling within multiple fixed time-intervals) was applied most frequently (*n* = 12), followed by the fixed sampling scheme (i.e. sampling at fixed times) (*n* = 9). Random sampling (i.e. completely random sampling) was chosen in three studies [34, 36, 55]. A mixed sampling approach was applied in three studies of which Lucassen et al*.* tested and compared both a fixed sampling and a semi-random sampling approach to assess overall dietary intake [22, 42, 57, 59]. Two studies applied different sampling schemes during the weekend compared to weekdays [22–33]. Sampling time windows were adapted to the daily structure of the study population, i.e. shifts of shift-workers, school hours of students or (self-reported) waking hours (Table 2). The sampling time window of the included studies started between 6 and 10 AM and ended between 8 PM and midnight. One study applied a 24-h sampling time window since the study population were nurses working in shifts [39].

#### Formulation of ESM questions

Different types of questions and phrasing of questions can be identified in the studies using ESM for dietary assessment. Two studies use indirect phrasing (i.e. ‘What were you doing?’) followed by multiple-choice answer options including i.e. physical activity, eating, rest [23–34]. Seven studies use direct phrasing (i.e. ‘Did you eat?’) which is applied both as real-time prompts (i.e. ‘Were you eating or drinking anything – in this moment?’) and as retrospective prompts (i.e. ‘Did you eat anything since the last signal?’) without specifying specific food consumption [22, 38, 40, 41, 45–48, 56, 58]. Thirteen studies use direct and specific phrasing regarding consumption of specified foods (i.e. ‘Did you eat any snacks or sugar sweetened beverages since the last signal?’) [35–37, 39, 43, 44, 50–55, 57]. The time period in retrospective prompts with direct phrasing varied. Ten studies assessed consumption since last signal, three studies during the past 2 h and one study during respectively the preceding 15 min, 1 h, 2.5 h, 3 h and 3.5 h [41, 42, 45–53, 56]. The MATCH study used two different retrospective time periods of which the first prompt of the day requested to report since waking up and the following prompts during the last 2 h [23–33]. Forman et al*.* used prompts which requested to report snack intake between the last prompt of the previous day and falling asleep and between waking up and receiving the first prompt [49]. The study of Bruening et al*.* combined both real-time prompts, to report what participants were doing the moment before receiving the prompt, and retrospective prompts to report what they were doing the past 3 h [34].

#### Formulation of ESM response options

Binary (i.e. yes or no) response options are provided in eleven studies followed by open field, a built in search function or multiple-choice bullets to specify type of food or drinks consumed in five studies [22, 35, 37, 38, 40–42, 45–48, 52, 53, 56, 58]. Food lists shown as response option to indicate food consumption were based on National Health Surveys, validated Food Frequency Questionnaires, other validated questionnaires, the National Food Composition Database or results from focus group discussions. Eight studies requested to indicate quantities of the foods consumed by open field (i.e. in grams or milliliters), Visual Analogical Scale (VAS) sliders (i.e. from zero to 100) or multiple-choice options (i.e. small, medium, large) [44–51, 54, 56, 57].

## Discussion

This review reveals that ESM has been applied to assess dietary intake in various research settings using different design approaches. However, most studies assessed consumption of specific foods only focusing on the foods of interest related to the research question. Especially snack consumption and, in general, unhealthy foods were the foods of interest for which ESM was used most often to measure its consumption. Due to its momentary nature, ESM may be especially suitable to measure these specific foods which are often (unconsciously) missed or underreported using traditional dietary assessment methods. Findings from our review show that ESM applied to assess dietary intake shows both features of 24-h dietary recalls (24HRs) and food frequency questionnaires (FFQ). Aside from the recall-based reporting and multiple choice assessment of specific foods, found in 24HRs and FFQs respectively, the ESM is a new methodology compared to traditional dietary assessment methods. ESM shows to lends itself well to assess the total dietary intake quantitatively as well albeit less explored yet according to our review. Moreover, most studies using ESM for dietary assessment were behavioral science research (i.e. psychological aspects of eating behavior) which highlights the novelty and need of ESM specifically designed for dietary assessment and research on diet-health associations.

### Recommendations to develop an Experience Sampling-based Dietary Assessment Method

The implementation of ESM will differ depending which health behavior is being measured and in which research field it is being applied [13, 60]. This section describes recommendations of the methodological implementation of ESM as an alternative dietary assessment methodology to measure total dietary intake quantitatively based on the findings of this review, recommendations of the open handbook for ESM by Myin-Germeys et al. and practices in traditional dietary assessment development [13].

#### Recommendations for study duration, ESM timing and frequency

All ESM study characteristics (study duration, sampling frequency, timing, recall period) are interrelated and cannot be evaluated individually.

ESM study duration (i.e. number of days) and sampling frequency (i.e. number of prompts per day) should be reconciled and should be inversely adapted to one another (i.e. short study duration allows for higher sampling frequency per day and vice versa) to maintain low burden and good feasibility.

Our review showed an ESM study duration of 7 days is most common however reporting fatigue might arise from day 4 onwards in case of high sampling frequency (i.e. fixed sampling every 2 h) similarly as experienced with food records [61].

Frequency and timing of ESM prompts should be adapted to waking hours covering the typical eating episodes of the target study population. Typically, studies used waking hours starting around 7 AM till 10 PM however a preliminary short survey can identify feasible and accurate waking hours of the target study population and allow to adapt accordingly.

Waking hours, and consequently sampling frequency, could be different on weekend days (i.e. more frequent, longer waking hours) as seen in some studies in our review. Short recall periods (i.e. last hours or previous day) are suggested to be better than longer recalls of weeks or months [62]. Aiming to obtain more accurate dietary intake data, lower recall bias and social desirability bias by reducing the awareness of being measured requests short recall periods of 1 up to 3.5 h, with a 2-h recall most commonly applied, as demonstrated by our review. In this way, ESM allow for near real-time measurements of dietary intake.

Furthermore, study duration, sampling frequency and timing should be adapted and differs when aiming to measure actual dietary intake or habitual dietary intake.

#### Recommendations ESM signaling technique for actual versus habitual dietary intake

Measuring actual dietary intake using an intensive prompting schedule can only be performed for short periods, preferably three to four days, due to the risk of responding fatigue as seen similarly in food records. As demonstrated by Lucassen et al. actual intake can be measured by ESM applying a fixed sampling approach which samples every time-window during the waking hours (i.e. sampling every 2 h between 7 AM and 10 PM on dietary intake during past 2 h) [58].

Habitual dietary intake can be measured by ESM applying a semi-random sampling approach which samples every time window during waking hours multiple times during a longer period (i.e. sampling three time-windows per day on dietary intake during past 2 h for two weeks until every time window is sampled three times) [58]. Measuring habitual dietary intake by ESM using a less intensive sampling frequency allows for a longer study duration (i.e. multiple weeks). Lastly, a combination of fixed and (semi-)random sampling schedules can be applied. Both in case of measuring actual and habitual dietary intake, it is recommended to compose a sampling schedule with time windows covering all waking hours to ensure all eating occasions could be sampled [12]. Additionally, the sampling schedule should cover weekend days next to week days to be able to sample the variability in dietary intake. More so, to capture variability of dietary intake several waves of ESM measurement periods could be implemented alternated with no-measurement periods. On the other hand, the application of multiple waves is associated with higher dropout rates especially with increased time in-between waves [13].

In conclusion, ESM signaling technique, frequency, timing, recall period and duration of sampling should be carefully adapted to one another to ensure accurate dietary intake data, low burden and optimal feasibility. As recommended by Myin-Germeys et al., a pilot study allows to evaluate all ESM design characteristics to obtain optimal data quality yet remain feasible [13].

#### Recommendations for ESM questions and response options

Questionnaires for ESM should be carefully developed and request methodological rigor [63]. As stated by Myin-Germeys et al., there are currently no specific guidelines on how to develop questionnaires for ESM [63]. However, according to our review most studies adapt existing questionnaires to implement in ESM research. Still, few studies in our review describe methodologically which or how adaptations are made to fit in the ESM format. First, a timeframe should be chosen on which the question will reflect. Although ESM is ideally consisting of questions on momentary variables, this is less suitable to measure dietary intake. As dietary intake does not continuously take place, momentary questions (i.e. What are you eating in this moment?) would lead to a large amount of missing data and, consequently, large measurement error on daily dietary intake estimations. Instead, time intervals lend itself better to assess dietary intake with ESM. The time interval on which the question reflects should be clearly stated (i.e. What did you eat during the last two hours?). As mentioned previously, in case of an interval contingent (semi-random) ESM approach, constitution of contiguous time intervals that cover the complete waking hour time frame (i.e. waking hours between 7 AM and 10 PM with semi-random ESM sampling by intervals of every two hours) is recommended to reduce risk of missing eating occasions [12]. Therefore, following the latter approach, it is most feasible to choose the same time frame on which the question reflects as the time intervals of the prompts (i.e. semi random sampling in time intervals of two hours with question ‘What did you eat since the last signal?’). The time frame on which the question reflects should be chosen based on expected events of dietary intake (i.e. every two or three hours) and depends on dietary habits of the target population which is culture specific. Myin-Germeys et al. recommend to keep questions short and to the point so it fits the screen of the mobile device and allows for quick response [63]. Furthermore, implicit assessments (i.e. Have you eaten since the last signal?) are recommended over explicit assessments (i.e. Did you eat fast food since the last signal?) to inhibit reactivity bias. Questionnaire length is important to consider as it is recommended to maintain a completion time of maximum three minutes to keep the burden low [63]. Although in traditional ESM research questionnaires up to 30 items are accepted, in the field of dietary assessment, this would equivalent a short FFQ and can be considered too burdensome when presented all at once at every prompt reducing compliance. Moreover, ESM research in the field of psychology, where it originated from, uses most often scales (i.e. Likert scale, visual scales) as respond options. Unlike many psychological variables (i.e. mood, emotions), dietary intake can be assessed quantitatively and precise which allows for more specific response options.

#### Recommendations to develop ESM sampling scheme based on FFQ or food record

**Fig. 2:**
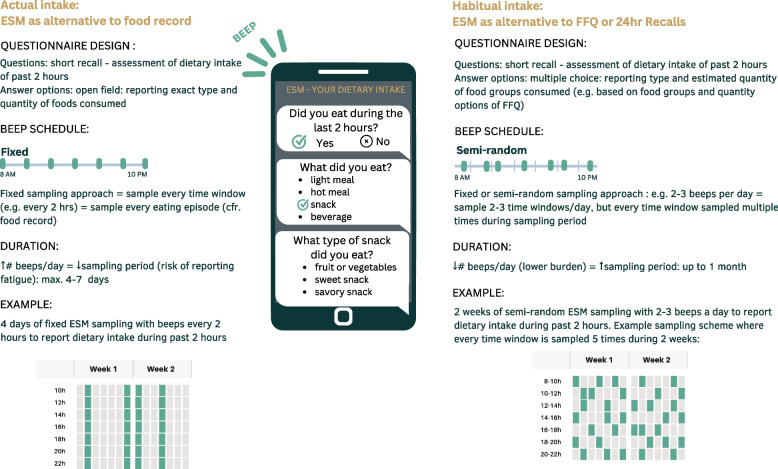
Recommendations to implement experience sampling for actual and habitual dietary assessment

Questions and respond options for ESM dietary assessment could be adapted from existing questionnaires as demonstrated in the studies of our review. In the field of dietary assessment, ESM could therefore be applied to validated dietary assessment questionnaires such as validated Food Frequency Questionnaires (FFQ’s) or (web-based) food records as proposed in Fig. 2.

Starting from the food record approach, a general open question (i.e. Did you eat anything since the last signal?) could be followed by a question to specify the consumed foods by an open field text box or food groups part originating from a National Food Consumption Database. Portion sizes of consumed foods could be provided by an open field text box with standard units (i.e. milliliters, grams) or common household measures (i.e. table spoons, glasses).

Starting from the FFQ approach, food groups assessed in FFQ’s could be regrouped to a limited number and questions reformulated to assess dietary intake in near real time to design ESM questionnaires. Consumption of all food groups could be assessed at each prompt or consumption of a different set of food groups could be assessed at each prompt. In the latter case, the study needs to be designed so that consumption of each food group is assessed at each interval multiple times to account for unanswered prompts with missing data. Moreover, ordering of questions on consumption of food groups need to be considered as the consumption of specific food groups might need to be assessed at the same prompt to reduce ambiguity (i.e. fried food consumption needs to be assessed before consumption of fast food to avoid response overlap). Asking the same set of questions at each prompt may feel repetitive but might reduce burden [63]. A control question can be added to assess careless responding.

### Application of ESM as alternative dietary assessment method in literature

Most studies used ESM to measure food consumption qualitatively (i.e. type of foods consumed) or semi-quantitatively (i.e. frequency of consumption of specific foods) as opposed to quantitatively (i.e. type and quantity of foods consumed) to serve the same purpose as traditional dietary assessment methods. Questions were most often formulated using direct phrasing and asking about consumption of specific foods since the last signal. Answers were most often binary (i.e. yes/no indicating consumption of specific foods since last signal) combined with options to specify type and/or frequency or amount of foods consumed. Only the studies of Jeffers et al*.* and Lucassen et al*.* apply ESM to measure total dietary intake quantitatively of which Lucassen et al*.* evaluated ESM specifically as an alternative methodology for dietary assessment [57, 58].

Although both event-contingent and signal-contingent approaches are being used for dietary assessment, signal-contingent ESM approaches might provide auspicious opportunities to overcome the limitations and biases of traditional dietary assessment methods [12]. The near-real time data collection combined with (semi-)random sampling shows potential to reduce the burden for the participant both by its low intensity of registering and by its shorter questions with easy respond options. Moreover, the (semi-)random sampling technique might make the participant less aware of being measured resulting in possibly lower social-desirability bias leading, together with the short recall period, to more accurate data. In combination with modern technology such as mobile applications feasibility could be enhanced as well. Adapting questions and response options from either a validated FFQ or food record allow for relatively easy implementation of ESM as alternative dietary assessment method for total dietary intake (i.e. all food groups). However, validity and reliability need to be evaluated in the target population, similarly as traditional dietary assessment methods.

The systematic review and meta-analysis of Perski et al*.* states to have reviewed the use of ESM to assess five key health behaviors including dietary behavior [60]. Similar to our findings, all four studies described by Perski et al*.* are assessing dietary intake through ESM of specific foods only instead of the total dietary pattern (i.e. all food groups). Moreover, Perski et al*.* included event-contingent sampling (i.e. registering dietary intake as it occurs) approaches as well. As highlighted by Schembre et al*.* event-contingent sampling entails similar limitations and biases such as social desirability bias and burden as the traditional dietary assessment methods [27]. Not surprising, as event-contingent sampling can be seen as a similar approach as the traditional food record and serves for this reason not the purpose of this review to define a new methodology to overcome the limitations of current traditional dietary assessment methods. Similarly, photo-based methodologies (i.e. using images as food diary by event-based sampling) are unlikely to overcome the limitations of traditional dietary assessment methods due to the large measurement error in estimation of portion sizes and types of foods and were for this reason excluded in our review [3]. Most importantly, the four included reviews on dietary behavior in the meta-analysis of Perski et al*.* lacked specific details on ESM design characteristics or methodological implication of ESM as alternative dietary assessment method. Still, the potential of ESM to obtain more accurate and reliable dietary data is highlighted together with the need for proper validation.

Altogether, the lacking details on important methodological aspects of ESM hinders drawing conclusions on common practices for implementation of ESM for quantitative dietary assessment. Nevertheless, Perski et al*.* emphasize the need for more elaboration on the methodological aspects in order to provide a summary of best practices on implementation of ESM for specific health behaviors including dietary behavior [60]. Our scoping review meets this need with key methodological recommendations for developing an experience sampling dietary assessment method for total dietary intake next to elaboration on commonly applied ESM design characteristics.

### Limitations and strengths

An important limitation of this scoping review is, inherent to scoping reviews, the less rigor search strategy and screening process. This will have resulted in an incomplete overview of studies describing ESM for dietary assessment. Still, this review has not the aim to assess outcomes of studies but rather evaluate how ESM can be applied for dietary assessment methodologically. Therefore, its strength lies in the assessment and description of ESM approaches specifically to provide insight in its use for quantitative dietary assessment as an alternative method for the traditional dietary assessment methods. To our knowledge, this has only been performed by Schembre et al. previously [12]. However, our scoping review is, to our knowledge, the first to describe practical recommendations for developing an ESM for total dietary assessment (i.e. all food groups). Additionally, only two studies were identified to have applied ESM for total dietary assessment. Consequently, limited evidence-based information was available in literature on the development of ESM characteristics (prompting schedule, duration, questionnaire design) for quantitative dietary assessment of total dietary intake. Nevertheless, studies on qualitative and semi-quantitative dietary assessment using ESM were described and form, together with the guidelines of Myin-Germeys et al., the base of practical guidelines of designing an ESM protocol for quantitative dietary assessment of total dietary intake. To our knowledge, this review is the first to discuss recommendations on the implementation of ESM for quantitative dietary assessment as an alternative for traditional dietary assessment methods.

## Conclusions

This review shows that ESM is increasingly being applied in research to measure dietary intake. However, few studies applied ESM to assess total dietary intake quantitatively with the same purpose of traditional dietary assessment methods. Still, the methodological characteristics of ESM show auspicious possibilities to overcome limitations of the classic dietary assessment methods. This paper provides guidance and is the starting point for the development of an Experience Sampling Dietary Assessment Method to assess total dietary intake quantitatively based on recent literature and theoretical background. Thorough evaluation and validation studies are needed to test the full potential of ESM as a feasible and accurate alternative for traditional dietary assessment methods.

### Supplementary Information

Supplementary Material 1.

## Data Availability

The data that support the findings of this manuscript are available from the corresponding author upon reasonable request. The review protocol can be downloaded at: KU Leuven repository.

## Abbreviations

EMA: Ecological Momentary Assessment
ESDAM: Experience Sampling-based Dietary Assessment Method
ESM: Experience Sampling Method
FFQ: Food Frequency Questionnaire
MATCH: Mother’s and Their Children’s Health
PRISMA-P: Preferred Reporting Items for Systematic review and Meta-Analysis Protocols
PRISMA-Scr: Preferred Reporting Items for Systematic review and Meta-Analysis extension for scoping reviews
SBB: Sugar Sweetened Beverages
VAS: Visual Analog Scale

## References

[1] Hebert JR, Hurley TG, Steck SE, Miller DR, Tabung FK, Peterson KE, et al. Considering the value of dietary assessment data in informing nutrition-related health policy. Adv Nutr. 2014;5(4):447–55. 25022993 10.3945/an.114.006189 PMC4085192

[2] Liang S, Nasir RF, Bell-Anderson KS, Toniutti CA, O’Leary FM, Skilton MR. Biomarkers of dietary patterns: a systematic review of randomized controlled trials. Nutr Rev. 2022;80(8):1856–95. 35211745 10.1093/nutrit/nuac009 PMC9263887

[3] Bingham S, Carroll RJ, Day NE, Ferrari P, Freedman L, Kipnis V, et al. Bias in dietary-report instruments and its implications for nutritional epidemiology. Public Health Nutr. 2002;5(6a):915–23. 12633516 10.1079/PHN2002383

[4] Kirkpatrick SI, Baranowski T, Subar AF, Tooze JA, Frongillo EA. Best Practices for Conducting and Interpreting Studies to Validate Self-Report Dietary Assessment Methods. J Acad Nutr Diet. 2019;119(11):1801–16. 31521583 10.1016/j.jand.2019.06.010

[5] Bennett DA, Landry D, Little J, Minelli C. Systematic review of statistical approaches to quantify, or correct for, measurement error in a continuous exposure in nutritional epidemiology. BMC Med Res Methodol. 2017;17(1):146. 28927376 10.1186/s12874-017-0421-6 PMC5606038

[6] Satija A, Yu E, Willett WC, Hu FB. Understanding nutritional epidemiology and its role in policy. Adv Nutr. 2015;6(1):5–18. 25593140 10.3945/an.114.007492 PMC4288279

[7] Kirkpatrick SI, Reedy J, Butler EN, Dodd KW, Subar AF, Thompson FE, et al. Dietary assessment in food environment research: a systematic review. Am J Prev Med. 2014;46(1):94–102. 24355678 10.1016/j.amepre.2013.08.015 PMC4558887

[8] The Science of Real-Time Data Capture: Self-Reports in Health Research: Oxford University Press; 2007. Available from: 10.1093/oso/9780195178715.001.0001.

[9] Verhagen SJ, Hasmi L, Drukker M, van Os J, Delespaul PA. Use of the experience sampling method in the context of clinical trials. Evid Based Ment Health. 2016;19(3):86–9. 27443678 10.1136/ebmental-2016-102418 PMC5040762

[10] Csikszentmihalyi M. Handbook of research methods for studying daily life: Guilford Press; 2011.

[11] Mestdagh M, Verdonck S, Piot M, Niemeijer K, Kilani G, Tuerlinckx F, et al. m-Path: an easy-to-use and highly tailorable platform for ecological momentary assessment and intervention in behavioral research and clinical practice. Front Digit Health. 2023;5:1182175. 37920867 10.3389/fdgth.2023.1182175 PMC10619650

[12] Schembre SM, Liao Y, O’Connor SG, Hingle MD, Shen SE, Hamoy KG, et al. Mobile Ecological Momentary Diet Assessment Methods for Behavioral Research: Systematic Review. JMIR Mhealth Uhealth. 2018;6(11): e11170. 30459148 10.2196/11170 PMC6280032

[13] Dejonckheere E, Erbas, Y. Designing an experience sampling study. In: Myin-Germeys I, Kuppens, P., editor. The open handbook of experience sampling methodology: A step-by-step guide to designing, conducting, and analyzing ESM studies: Center for Research on Experience Sampling and Ambulatory Methods Leuven; 2021. p. 33–70.

[14] Arksey H, O’Malley L. Scoping Studies: Towards a Methodological Framework. International Journal of Social Research Methodology: Theory & Practice. 2005;8:19–32. 10.1080/1364557032000119616

[15] Levac D, Colquhoun H, O’Brien KK. Scoping studies: advancing the methodology. Implement Sci. 2010;5(1):69. 20854677 10.1186/1748-5908-5-69 PMC2954944

[16] Moher D, Shamseer L, Clarke M, Ghersi D, Liberati A, Petticrew M, et al. Preferred reporting items for systematic review and meta-analysis protocols (PRISMA-P) 2015 statement. Syst Rev. 2015;4(1):1. 25554246 10.1186/2046-4053-4-1 PMC4320440

[17] JBI JBI. [cited 2022 October 28th]. Available from: https://jbi.global/scoping-review-network/resources.

[18] Tricco AC, Lillie E, Zarin W, O’Brien KK, Colquhoun H, Levac D, et al. PRISMA Extension for Scoping Reviews (PRISMA-ScR): Checklist and Explanation. Ann Intern Med. 2018;169(7):467–73. 30178033 10.7326/M18-0850

[19] Khangura S, Konnyu K, Cushman R, Grimshaw J, Moher D. Evidence summaries: the evolution of a rapid review approach. Syst Rev. 2012;1(1):10. 22587960 10.1186/2046-4053-1-10 PMC3351736

[20] Khangura S, Polisena J, Clifford TJ, Farrah K, Kamel C. RAPID REVIEW: AN EMERGING APPROACH TO EVIDENCE SYNTHESIS IN HEALTH TECHNOLOGY ASSESSMENT. Int J Technol Assess Health Care. 2014;30(1):20–7. 24451157 10.1017/S0266462313000664

[21] Ganann R, Ciliska D, Thomas H. Expediting systematic reviews: methods and implications of rapid reviews. Implement Sci. 2010;5(1):56. 20642853 10.1186/1748-5908-5-56 PMC2914085

[22] Grenard JL, Stacy AW, Shiffman S, Baraldi AN, MacKinnon DP, Lockhart G, et al. Sweetened drink and snacking cues in adolescents: a study using ecological momentary assessment. Appetite. 2013;67:61–73. 23583312 10.1016/j.appet.2013.03.016 PMC3677830

[23] Dunton GF, Dzubur E, Huh J, Belcher BR, Maher JP, O’Connor S, et al. Daily Associations of Stress and Eating in Mother-Child Dyads. Health Educ Behav. 2017;44(3):365–9. 27531169 10.1177/1090198116663132

[24] Dunton GF, Liao Y, Dzubur E, Leventhal AM, Huh J, Gruenewald T, et al. Investigating within-day and longitudinal effects of maternal stress on children’s physical activity, dietary intake, and body composition: Protocol for the MATCH study. Contemp Clin Trials. 2015;43:142–54. 25987483 10.1016/j.cct.2015.05.007 PMC4861058

[25] O’Connor SG, Ke W, Dzubur E, Schembre S, Dunton GF. Concordance and predictors of concordance of children’s dietary intake as reported via ecological momentary assessment and 24 h recall. Public Health Nutr. 2018;21(6):1019–27. 29352820 10.1017/S1368980017003780 PMC10261495

[26] O’Connor SG, Koprowski C, Dzubur E, Leventhal AM, Huh J, Dunton GF. Differences in Mothers’ and Children’s Dietary Intake during Physical and Sedentary Activities: An Ecological Momentary Assessment Study. J Acad Nutr Diet. 2017;117(8):1265–71. 28392348 10.1016/j.jand.2017.02.012 PMC5534183

[27] Liao Y, Schembre SM, O’Connor SG, Belcher BR, Maher JP, Dzubur E, et al. An Electronic Ecological Momentary Assessment Study to Examine the Consumption of High-Fat/High-Sugar Foods, Fruits/Vegetables, and Affective States Among Women. J Nutr Educ Behav. 2018;50(6):626–31. 29573964 10.1016/j.jneb.2018.02.003 PMC5995648

[28] Mason TB, Naya CH, Schembre SM, Smith KE, Dunton GF. Internalizing symptoms modulate real-world affective response to sweet food and drinks in children. Behav Res Ther. 2020;135: 103753. 33049549 10.1016/j.brat.2020.103753 PMC7793613

[29] Mason TB, O’Connor SG, Schembre SM, Huh J, Chu D, Dunton GF. Momentary affect, stress coping, and food intake in mother-child dyads. Health Psychol. 2019;38(3):238–47. 30762403 10.1037/hea0000714 PMC6436946

[30] Mason TB, Smith KE, Dunton GF. Maternal parenting styles and ecological momentary assessment of maternal feeding practices and child food intake across middle childhood to early adolescence. Pediatr Obes. 2020;15(10): e12683. 32543051 10.1111/ijpo.12683 PMC8862542

[31] Do B, Yang CH, Lopez NV, Mason TB, Margolin G, Dunton GF. Investigating the momentary association between maternal support and children’s fruit and vegetable consumption using ecological momentary assessment. Appetite. 2020;150: 104667. 32173569 10.1016/j.appet.2020.104667 PMC7164557

[32] Naya CH, Chu D, Wang WL, Nicolo M, Dunton GF, Mason TB. Children’s Daily Negative Affect Patterns and Food Consumption on Weekends: An Ecological Momentary Assessment Study. J Nutr Educ Behav. 2022;54(7):600–9. 35644784 10.1016/j.jneb.2022.02.007 PMC9276542

[33] Lopez NV, Lai MH, Yang CH, Dunton GF, Belcher BR. Associations of Maternal and Paternal Parenting Practices With Children’s Fruit and Vegetable Intake and Physical Activity: Preliminary Findings From an Ecological Momentary Study. JMIR Form Res. 2022;6(8): e38326. 35947425 10.2196/38326 PMC9403822

[34] Bruening M, van Woerden I, Todd M, Brennhofer S, Laska MN, Dunton G. A Mobile Ecological Momentary Assessment Tool (devilSPARC) for Nutrition and Physical Activity Behaviors in College Students: A Validation Study. J Med Internet Res. 2016;18(7): e209. 27465701 10.2196/jmir.5969 PMC4980553

[35] Piontak JR, Russell MA, Danese A, Copeland WE, Hoyle RH, Odgers CL. Violence exposure and adolescents’ same-day obesogenic behaviors: New findings and a replication. Soc Sci Med. 2017;189:145–51. 28768573 10.1016/j.socscimed.2017.07.004 PMC5907915

[36] Campbell KL, Babiarz A, Wang Y, Tilton NA, Black MM, Hager ER. Factors in the home environment associated with toddler diet: an ecological momentary assessment study. Public Health Nutr. 2018;21(10):1855–64. 29526170 10.1017/S1368980018000186 PMC10261025

[37] Cummings JR, Mamtora T, Tomiyama AJ. Non-food rewards and highly processed food intake in everyday life. Appetite. 2019;142: 104355. 31291596 10.1016/j.appet.2019.104355 PMC6717547

[38] Maher JP, Harduk M, Hevel DJ, Adams WM, McGuirt JT. Momentary Physical Activity Co-Occurs with Healthy and Unhealthy Dietary Intake in African American College Freshmen. Nutrients. 2020;12(5):1360. 10.3390/nu12051360 PMC7285035 32397433

[39] Lin TT, Park C, Kapella MC, Martyn-Nemeth P, Tussing-Humphreys L, Rospenda KM, et al. Shift work relationships with same- and subsequent-day empty calorie food and beverage consumption. Scand J Work Environ Health. 2020;46(6):579–88. 32449516 10.5271/sjweh.3903 PMC7737792

[40] Yong JYY, Tong EMW, Liu JCJ. When the camera eats first: Exploring how meal-time cell phone photography affects eating behaviours. Appetite. 2020;154:104787. 10.1016/j.appet.2020.104787 32579971

[41] Goldstein SP, Hoover A, Evans EW, Thomas JG. Combining ecological momentary assessment, wrist-based eating detection, and dietary assessment to characterize dietary lapse: A multi-method study protocol. Digit Health. 2021;7:2055207620988212. 10.1177/2055207620988212 PMC7863144 33598309

[42] Chmurzynska A, Mlodzik-Czyzewska MA, Malinowska AM, Radziejewska A, Mikołajczyk-Stecyna J, Bulczak E, et al. Greater self-reported preference for fat taste and lower fat restraint are associated with more frequent intake of high-fat food. Appetite. 2021;159:105053. 10.1016/j.appet.2020.105053 33248190

[43] Barchitta M, Maugeri A, Favara G, Magnano San Lio R, Riela PM, Guarnera L, et al. Development of a Web-App for the Ecological Momentary Assessment of Dietary Habits among College Students: The HEALTHY-UNICT Project. Nutrients. 2022;14(2):330. 10.3390/nu14020330 PMC8779738 35057511

[44] Spook JE, Paulussen T, Kok G, Van Empelen P. Monitoring dietary intake and physical activity electronically: feasibility, usability, and ecological validity of a mobile-based Ecological Momentary Assessment tool. J Med Internet Res. 2013;15(9): e214. 24067298 10.2196/jmir.2617 PMC3785990

[45] Wouters S, Jacobs N, Duif M, Lechner L, Thewissen V. Affect and between-meal snacking in daily life: the moderating role of gender and age. Psychol Health. 2018;33(4):555–72. 28934860 10.1080/08870446.2017.1380813

[46] Wouters S, Jacobs N, Duif M, Lechner L, Thewissen V. Negative affective stress reactivity: The dampening effect of snacking. Stress Health. 2018;34(2):286–95. 28971580 10.1002/smi.2788 PMC5900576

[47] Wouters S, Thewissen V, Duif M, Lechner L, Jacobs N. Assessing Energy Intake in Daily Life: Signal-Contingent Smartphone Application Versus Event-Contingent Paper and Pencil Estimated Diet Diary. Psychol Belg. 2016;56(4):357–69. 30479445 10.5334/pb.339 PMC5854103

[48] Wouters S, Thewissen V, Duif M, van Bree RJ, Lechner L, Jacobs N. Habit strength and between-meal snacking in daily life: the moderating role of level of education. Public Health Nutr. 2018;21(14):2595–605. 29808785 10.1017/S1368980018001283 PMC6141992

[49] Forman EM, Shaw JA, Goldstein SP, Butryn ML, Martin LM, Meiran N, et al. Mindful decision making and inhibitory control training as complementary means to decrease snack consumption. Appetite. 2016;103:176–83. 27083129 10.1016/j.appet.2016.04.014

[50] Richard A, Meule A, Reichenberger J, Blechert J. Food cravings in everyday life: An EMA study on snack-related thoughts, cravings, and consumption. Appetite. 2017;113:215–23. 28249745 10.1016/j.appet.2017.02.037

[51] Richard A, Meule A, Blechert J. Implicit evaluation of chocolate and motivational need states interact in predicting chocolate intake in everyday life. Eat Behav. 2019;33:1–6. 30738363 10.1016/j.eatbeh.2019.01.006

[52] Zenk SN, Horoi I, McDonald A, Corte C, Riley B, Odoms-Young AM. Ecological momentary assessment of environmental and personal factors and snack food intake in African American women. Appetite. 2014;83:333–41. 25239402 10.1016/j.appet.2014.09.008 PMC4376474

[53] Ghosh Roy P, Jones KK, Martyn-Nemeth P, Zenk SN. Contextual correlates of energy-dense snack food and sweetened beverage intake across the day in African American women: An application of ecological momentary assessment. Appetite. 2019;132:73–81. 30261234 10.1016/j.appet.2018.09.018

[54] Ortega A, Bejarano CM, Hesse DR, Reed D, Cushing CC. Temporal discounting modifies the effect of microtemporal hedonic hunger on food consumption: An ecological momentary assessment study. Eat Behav. 2022;48: 101697. 36527988 10.1016/j.eatbeh.2022.101697

[55] Boronat A, Clivillé-Pérez J, Soldevila-Domenech N, Forcano L, Pizarro N, Fitó M, et al. Mobile Device-assisted Dietary Ecological Momentary Assessments for the Evaluation of the Adherence to the Mediterranean Diet in a Continuous Manner. J Vis Exp. 2021(175). 10.3791/62161 34633362

[56] de Rivaz R, Swendsen J, Berthoz S, Husky M, Merikangas K, Marques-Vidal P. Associations between Hunger and Psychological Outcomes: A Large-Scale Ecological Momentary Assessment Study. Nutrients. 2022;14(23). 10.3390/nu14235167 PMC9736756 36501197

[57] Lucassen DA, Brouwer-Brolsma EM, Slotegraaf AI, Kok E, Feskens EJM. DIetary ASSessment (DIASS) Study: Design of an Evaluation Study to Assess Validity, Usability and Perceived Burden of an Innovative Dietary Assessment Methodology. Nutrients. 2022;14(6). 10.3390/nu14061156. 10.3390/nu14061156 PMC8949267 35334813

[58] Jeffers AJ, Mason TB, Benotsch EG. Psychological eating factors, affect, and ecological momentary assessed diet quality. Eat Weight Disord. 2020;25(5):1151–9. 31388844 10.1007/s40519-019-00743-3

[59] Lucassen DA, Brouwer-Brolsma EM, Boshuizen HC, Mars M, de Vogel-Van den Bosch J, Feskens EJ. Validation of the smartphone-based dietary assessment tool “Traqq” for assessing actual dietary intake by repeated 2-h recalls in adults: comparison with 24-h recalls and urinary biomarkers. Am J Clin Nutr. 2023;117(6):1278–87. 37054887 10.1016/j.ajcnut.2023.04.008

[60] Perski O, Keller J, Kale D, Asare BY, Schneider V, Powell D, et al. Understanding health behaviours in context: A systematic review and meta-analysis of ecological momentary assessment studies of five key health behaviours. Health Psychol Rev. 2022;16(4):576–601. 35975950 10.1080/17437199.2022.2112258 PMC9704370

[61] Thompson FE, Subar AF. Chapter 1 - Dietary Assessment Methodology. In: Coulston AM, Boushey CJ, Ferruzzi MG, Delahanty LM, editors. Nutrition in the Prevention and Treatment of Disease (Fourth Edition): Academic Press; 2017. p. 5–48.

[62] Shiffman S, Balabanis MH, Gwaltney CJ, Paty JA, Gnys M, Kassel JD, et al. Prediction of lapse from associations between smoking and situational antecedents assessed by ecological momentary assessment. Drug Alcohol Depend. 2007;91(2-3):159–68. 10.1016/j.drugalcdep.2007.05.017 PMC2244586 17628353

[63] Eisele G, Kasanova Z, Houben M. Questionnaire design and evaluation. In: Myin-Germeys I, Kuppens, P., editor. The open handbook of Experience Sampling Methodology: A step-by-step guide to designing, conducting, and analyzing ESM studies. Center for Research on Experience Sampling and Ambulatory Methods Leuven; 2021. p. 71–90.

