# Supplementary material for: Experience Sampling as a dietary assessment method: a scoping review towards implementation
Source: Int J Behav Nutr Phys Act. 2024 Aug 27;21:94. doi: 10.1186/s12966-024-01643-1 (PMC11350948; doi:10.1186/s12966-024-01643-1)
Supplement: Supplementary file 1 — Supplementary Material 1. [file 12966_2024_1643_MOESM1_ESM.docx]

SUPPLEMENTARY MATERIAL: Search string

1. PubMed Search 09/02/2024:

Search string: (("experience sampling"[Title/Abstract] AND 2012/01/01:2022/11/09[Date - Publication]) OR ("ecological momentary assessment"[Title/Abstract] AND 2012/01/01:2022/11/09[Date - Publication])) AND ("nutrition assessment"[MeSH Terms] OR "dietary assessment"[Title/Abstract] OR "food intake"[Title/Abstract] OR "nutritional assessment"[Title/Abstract])

1. Web of Science Search 09/02/2024:

Search string: (Experience sampling OR Ecological momentary assessment) AND (nutrition assessment OR dietary assessment OR food intake OR nutritional assessment) Limitation: 01-01-2012 till 09-02-2024, citation topics meso: nutrition & dietetics
